# Would patients undergo postoperative follow-up by using a smartphone application?

**DOI:** 10.1186/s12893-020-00889-3

**Published:** 2020-10-07

**Authors:** Julian Scherer, Frank Keller, Hans-Christoph Pape, Georg Osterhoff

**Affiliations:** 1 Department of Traumatology, University Hospital Zurich, Raemistrasse 100, 8091 Zurich, Switzerland; 2 Orthozentrum Rosenheim, Äußere Münchener Straße 94, 83026 Rosenheim, Germany; 3 Department of Orthopaedics, Trauma Surgery and Plastic Surgery, University Hospital of Leipzig, Liebigstr. 20, 04103 Leipzig, Germany

**Keywords:** mHealth, eHealth, Smartphone application, Follow-up, Big data

## Abstract

**Background:**

eHealth applications have been proposed as an alternative to monitor patients in frequent intervals or over long distances. The aim of this study was to assess whether patients would accept an application on their smartphone to be monitored by their physicians.

**Methods:**

During September 2017 and December 2017 a survey amongst smartphone users was conducted via paper and web-based questionnaires.

**Results:**

More than half of the 962 participants (54%) were older than 55 years of age. The majority of the participants (68.7%) would accept a follow-up by a smartphone application obtaining personal healthcare data. 72.6% of all patients older than 55 years of age would use the application. The most prevalent reason against installing the application was data protection. Patients being currently treated in an orthopaedic practice and pedestrians were more eager to accept a follow-up by a mobile app than participants from social media.

**Conclusion:**

The majority of participants would accept a mobile application, collecting personal health-related data for postoperative follow-up, and saw a direct benefit for the patient in such an application.

## Background

Today, the majority of people, including the elderly living in developed countries have access to a smartphone. In the USA, 42% of all people who are over the age of 65, own a smartphone [1] and in the European Union, 23% of people who are older than 64 years used a smartphone to access the internet in 2016 [2]. Smartphone health applications have become popular on several common smartphone operating systems. In a survey of 1604 smartphone users in the USA, 58% had downloaded a health-related application on their smartphones in 2015 [3]. eHealth applications can be used as a cost-effective and comfortable instrument to transfer patients’ medical data to their treating physicians, even over long distances. They can be easily implemented in order to obtain patient-related recovering data after e.g. orthopaedic surgery [4]. Telemedicine can lower consultation duration and distance travelled by the patient significantly and therefore can help patients accessing the health care system [5, 6] and up to 60% of orthopaedic surgeons in the USA would implement telemedicine in orthopaedics as a follow-up tool in patients who live far away [7]. Furthermore, there are several other studies of various surgical disciplines showing the benefit of telemedical surgical aftercare and follow-up [6, 8–12].

However, little is known as to, whether patients would accept to have their personal health-related data recorded and transferred to the treating surgeon via eHealth applications.

The aim of this study was to conduct a survey investigating the willingness of individuals from different age groups to use a smartphone application to let surgeons monitor the postoperative follow-up interval. The study also investigated patients’ concerns and appreciation about the mentioned hypothetical follow-up setting.

## Methods

### Patients and survey design

A web-based questionnaire was created using (Google Forms®) which was shared randomly via social media (Facebook®) to acquire participants. In addition, the same questionnaire was handed out on paper, to patients of an orthopaedic practice of one of the authors (FK, Rosenheim, Germany) and to pedestrians in the city center of Zurich, Switzerland. The questionnaire was designed exclusively for this survey. The survey was conducted from September 2017 to December 2017. The only inclusion criteria were the possession and daily usage of a smartphone. By answering the questionnaire, participants gave consent to the use of the data that they had provided. The institutional ethics committee (Clinical Trial Center of the University Hospital of Zurich) ruled that no formal ethics approval was required.

The questionnaire (Appendix 1) described the following scenario: The patient is being operated on by a surgical team. They are offered to install a smartphone application on the patient’s mobile phone to monitor the long-term outcome of the operation and to intervene in the event of any complications, in the sense of early detection. The application was described with its specific properties: 1. data transfer to a protected hospital server, synchronized of the app’s profile data and the hospital’s data 2. no data access for third parties other than anonymized for research, 3. acquisition only of direct motion data from the phone (e.g. step count), no sensitive data (e.g. geo-data via GPS), 5. the app would ask to respond to a maximum of two questions maximum once a day (e.g. “did you forget your phone?” in the case no movement was recorded, or “are you sick?”), 6. in case of data irregularities, the treating surgeon would contact the patient, 7. the patient has no influence on the type of data acquired (no manipulation of the data possible), 8. the patient can read-out only absolute data but no interpretation (exclusion of anxiety, hypochondria), 9. the app is for free.

Participants were then asked whether they would install the app or not, and to choose from a number of reasons for their response. In addition, the participants’ baseline characteristics including age, sex and profession were obtained. At the end of the questionnaire, they were able to leave a comment on the survey.

### Statistical analysis

Further statistical analysis was done by the use of SPSS® Statistics Desktop 24.0 for Mac (SPSS, Chicago, Illinois, USA). Data is presented as frequencies (n) and means with the standard deviation (SD). To assess differences in means between the two groups, an independent-samples t-test was used for the normally distributed continuous data and a Chi-Square test for categorical data. A subgroup analysis was performed for the age (group < 30 years, group 30 to 55 years, group > 55 years), gender and presence of a medical background. The level of statistical significance was set at *p* < 0.05. A second subgroup analysis was performed for the source of answered questionnaires (orthopaedic practice, pedestrians and social media).

## Results

**Fig. 1:**
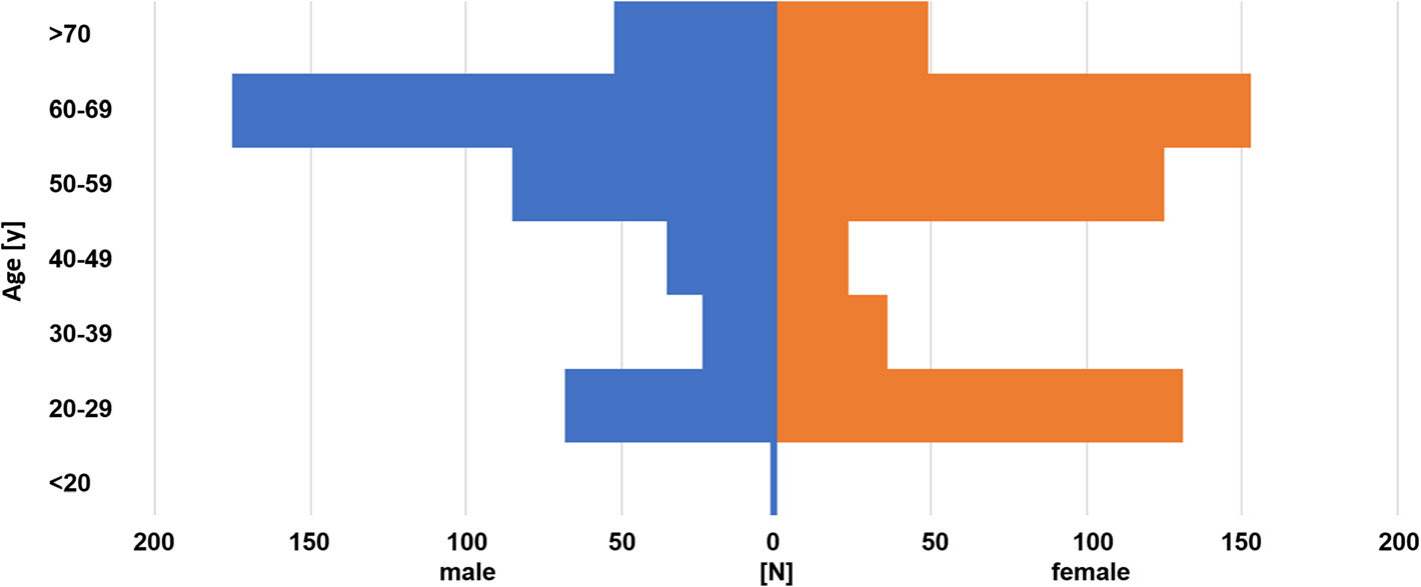
Age and gender distribution

In total, 962 participants (female: 521, 54.2% with a mean age of 51.7 years (SD 17.4 years) were included. (Fig. 1) Most participants (54.0%) were older than 55 years of age. Of the total of 962 participants, 527 (54.8%) questionnaires were obtained in the orthopaedic practice in Rosenheim, Germany, 86 (8.9%) questionnaires were obtained by asking random pedestrians in the city center of Zurich and 349 (36.3%) questionnaires were obtained via random sharing on social media (Facebook®). The mean age of the orthopeadic practice group was 60.4 years (SD 11.1 years), in the social media group, we found a mean age of 35.7 years (SD 15.3 years) and the mean age of the pedestrians was 63.8 years (SD 5.1 years).

**Fig. 2:**
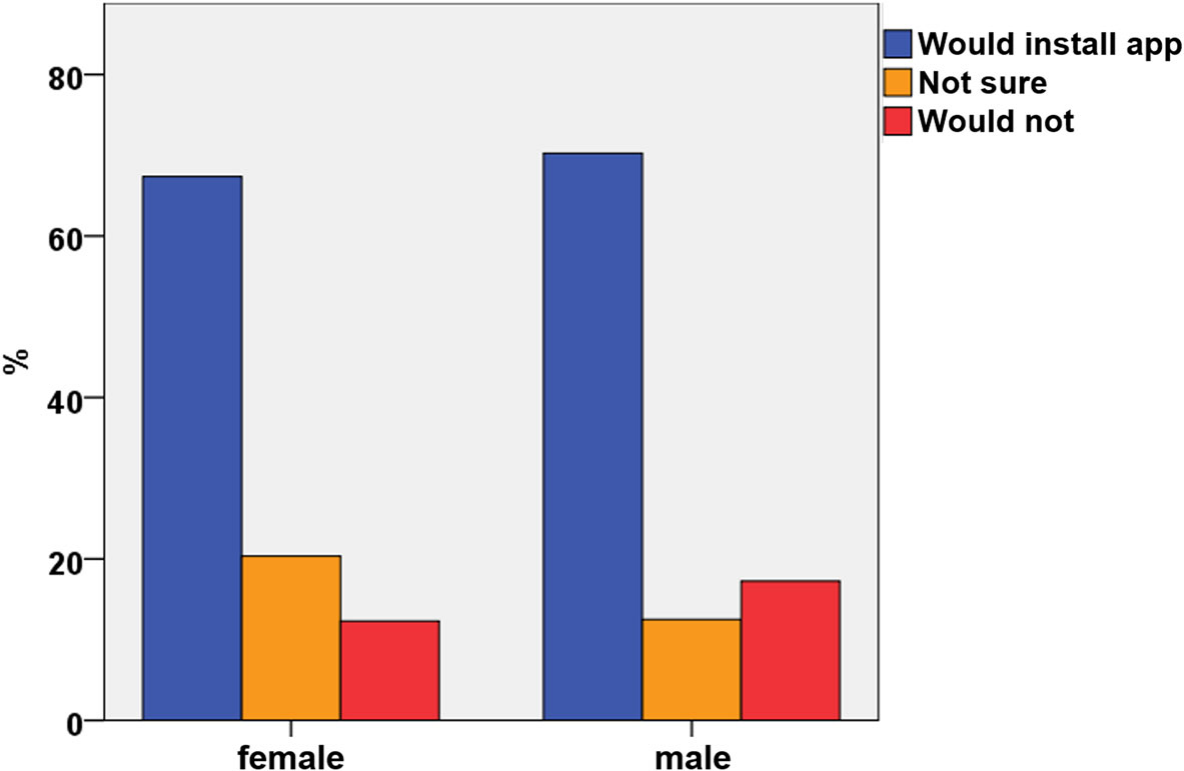
Acceptance of a smartphone application by gender

The majority of all participants (68.7%) would accept a follow-up by a smartphone application obtaining personal healthcare data compared to 14.6% who would not; 16.7% were unsure. Male participants were more likely to install an app on their phone (*p* = 0.001, Fig. 2).

**Fig. 3:**
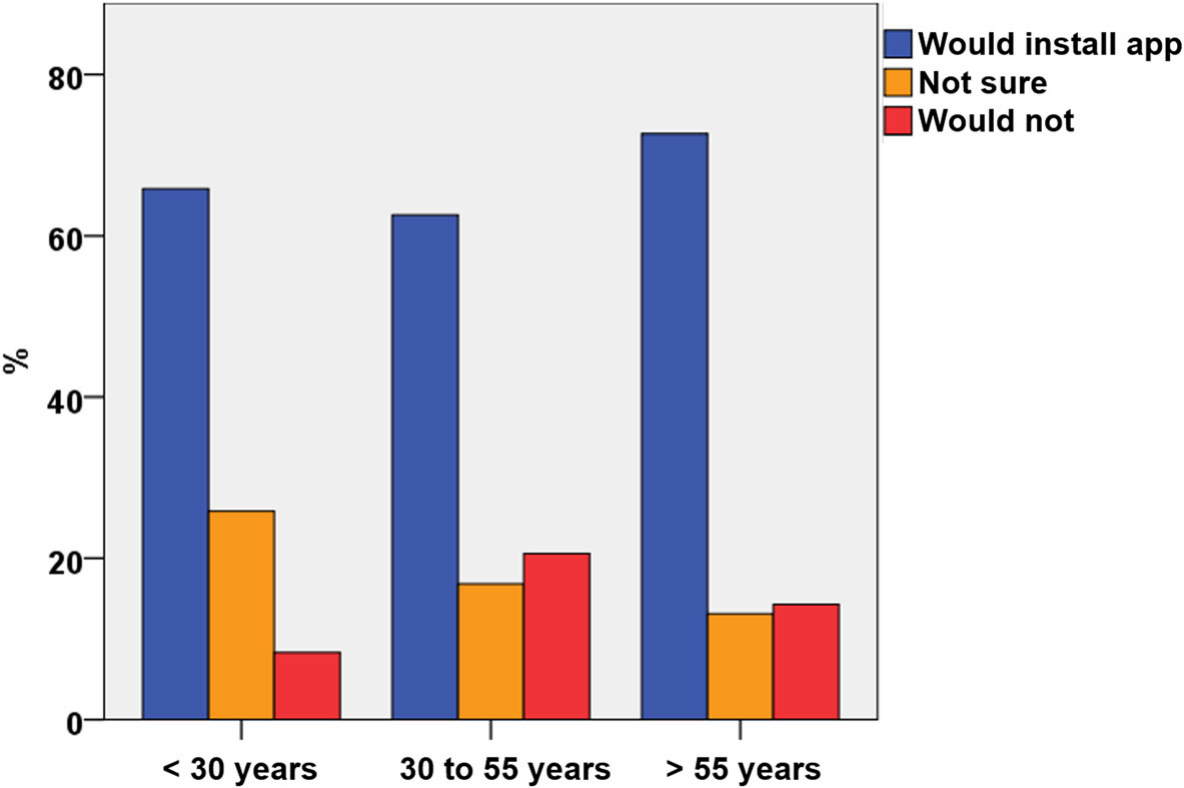
Acceptance of a smartphone application by age

Participants older than 55 years of age were more likely to accept the idea of a follow-up using a mobile application (72.6%), when compared to patients younger than 55 years of age (64.1%, *p* = 0.004). The lowest acceptance rate was seen in the group between 30 to 55 years of age. (Fig. 3).

**Fig. 4:**
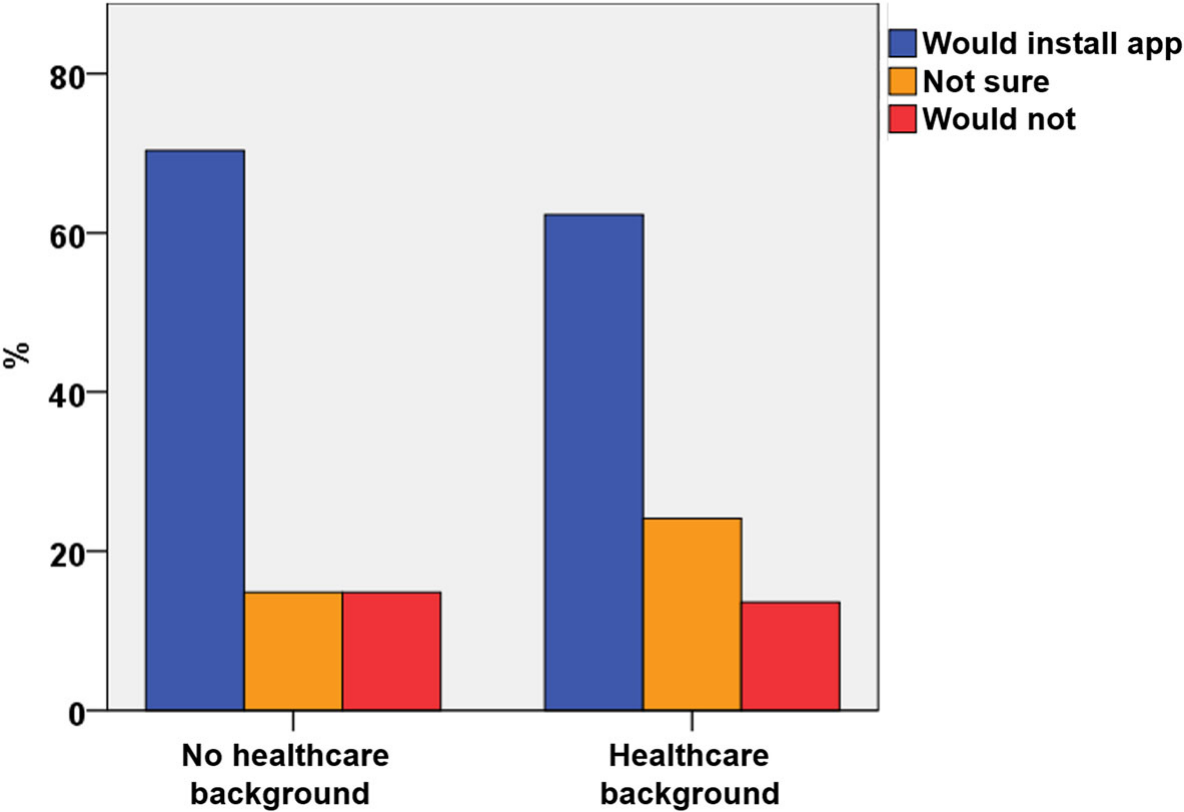
Acceptance of a smartphone application by occupational background

Regarding their occupation, 20.6% of the participants had a healthcare background (70.4% female and 29.6% male). Within this group, 62.3% would install an app for mobile follow-up compared to participants without a healthcare occupation (70.4%, *p* = 0.007, Fig. 4).

**Fig. 5:**
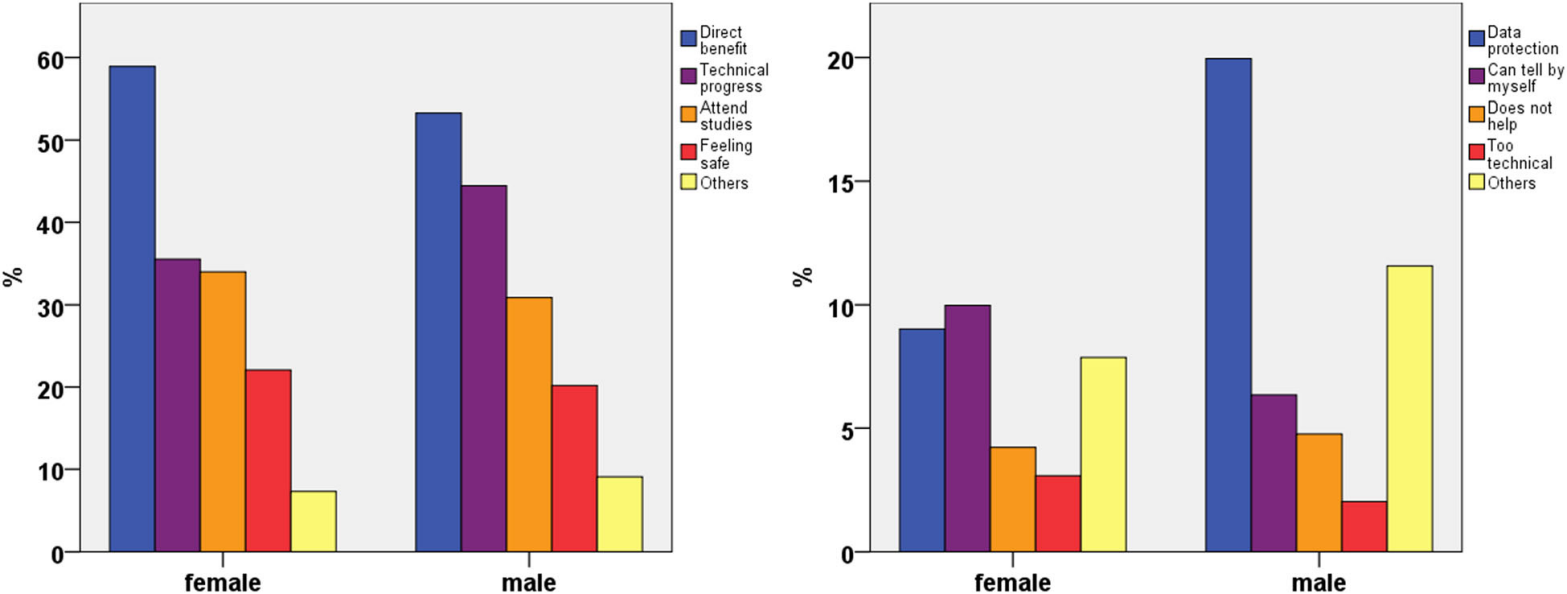
Agreement with statements in favor of and against using a smartphone application for postoperative follow-up

The statement most frequently agreed with in favor of using the application was “I have a direct benefit through the app, in the sense of early detection” (all: 56.3%; among participants accepting the application: 72.9%), followed by “I support technology in medicine” (39.6%) and “I like to take part in studies to support medical progress” (32.5%, Fig. 5).

The statement against using the application most frequently agreed with was “data protection” (all: 14%; among participants not accepting the application: 51.4%), followed by “others” (9.6%) and “I can tell by myself when something is wrong” (8.3%, Fig. 5). Among all age groups, most participants agreed with “I have direct benefit through the app, in the sense of early detection” (group < 30: 60.0%, group 30 to 55: 50.0%, group > 55: 57.8%; (Fig. 5).

There were no significant differences in statements in favor of the application by gender: Both, most female (58.9%) and male (53.3%) participants preferred “I have direct benefit through the application, in the sense of early detection” as a reason to install the application. More male than female participants were concerned about “data protection” (males 20%; females 9%; *p* < 0.001). The main reason against the application in the female cohort was “I can tell by myself when something is wrong” (10%).

The majority of the participants who underwent the survey in the orthopaedic practice (77.4%) would install the proposed mobile app, followed by 68.6% in the group of pedestrians and 55.6% in the social media group (*p =* < 0.001). The statement most frequently agreed with in favor of using the application was “I have a direct benefit through the app, in the sense of early detection” (orthopaedic practice: 58.6%, social media: 51.9% and pedestrians: 60.5%, *p* = 0.102). The second most frequently stated advantage was “I like to take part in studies to support medical progress” with no statistical difference between the assessed groups (*p* = 0.175). “I feel safe being constantly monitored” was stated significantly more often in the group of pedestrians (27.9%) than in the group of the orthopaedic practice (24.5%) and the social media group (14.6) (*p* = 0.001). Pedestrians and participants from the orthopaedic practice (44.2 and 43.5%) stated significantly more often that they “support technology in medicine” than participants from the social media group (32.7%) (*p* = 0.004).

In all three groups, the most stated reason against using the app was “data security” (orthopaedic practice: 13.9%, social media: 15.2%, pedestrians: 10.5%, *p* = 0.520). There were no significant differences between the groups regarding other disadvantages of the imaginary mobile application.

## Discussion

The purpose of the present study was to identify whether surgical patients would accept a smartphone application to let their surgeon monitor postoperative follow-up and recovery.

The survey indicated that most of the people (68.7%) would install a health application on their mobile phone to obtain data for clinical use and to observe the patients` long-term outcome after surgery. A similar survey among patients with chronic diseases revealed that 60% would be interested in telemedicine using a smartphone [13]. Interestingly, there was a significantly higher acceptance rate in the group of participants more than 55 years of age. Also, the participants from the pedestrians group as well as from the orthopaedic practice group with a mean age more than 24 years higher compared to their counterparts from the social media group, had significant higher acceptance rates in this survey. Furthermore, these two groups stated significantly more often that they support technology in medicine. This is consistent with recent reports that elderly people are very interested in the use of novel technology [14]. Aged people are more likely to undergo surgery and more likely to be affected by impaired mobility [15]. Hence, they have a special interest in technologies that allow for a reduction of frequent follow-up consultations. For them, “the direct benefit in the sense of early detection” seemed to overweigh concerns about “data protection”. In contrast, middle-aged men with a health care background were the most hesitant to be monitored by a mobile application. There is a general fear of big data and loss of personal information [16] and 45% of internet users are afraid of misuse of personal data, which is consistent with our findings, that participants from the social media group were more afraid of data security, had less interest in supporting technology in medicine and were less eager to use the proposed mobile application [17]. In the comments section of the questionnaire, many people stressed that, especially health insurance companies, should not be able to have access to the application data. This is consistent with the fact that participants with a medical background were more critical about the application as they may know better potential ways of data misuse in the context of health care.

This study has its limitations. Firstly, the mobile application of interest in this survey does not exist in reality and we cannot be sure whether an application with the stated specifics would actually be practicable (e.g. safety with regard to potential access of third parties). Secondly, even though age and gender were evenly distributed, more than half of the participants were actual patients under or expecting orthopaedic treatment which could cause a bias to the representativeness of the surveyed cohort. However, the authors tried to minimize this bias: the paper-questionnaire was handed out by the doctors` assistants in the mentioned orthopaedic group practice and the paper-questionnaire did not state any affiliation with the treating physician. Unfortunately, distance to practice or hospital and educational level were not questioned as we wanted to keep the questionnaire short and with a focus on the general acceptance of such an application.

A follow-up using a smartphone application may be very advantageous in clinical daily routine for both, the patients and the physicians. Patients’ fear of non-recognized post-surgical complications could be lowered, and unnecessary consultations and costs could be avoided. In order to avoid legal issues and to make sure that adverse after care events would not be missed, an automated system has to be developed which alerts the treating physician sufficiently. Furthermore, in times when direct physical consultations should be avoided, unnecessary contact could be reduced. The standardized collection of outcome data could be used for quality assessment and research. This would, eventually, lead to improved health care for all. In the light of recent reports on big data leakage and the flaws in medical device security [18], concerns about data protection are justified and need to be addressed before implementation of such a tool. As also shown in this study, acceptance of any mobile application that collects personal health-related data is directly linked to data protection.

## Conclusion

The majority of the participants in this survey among smartphone users would accept a mobile application collecting personal health-related data for postoperative follow-up. Most participants saw a direct benefit for the patient in such an application. Major concerns included data protection. In order to establish such a tool, the most important next step would be to address concerns about data safety and security as well as identification of socioeconomic factors.

## Supplementary information

**Additional file 1.**

## Data Availability

Data supporting this manuscript can be requested from the corresponding author.
