# Supplementary material for: Would patients undergo postoperative follow-up by using a smartphone application?
Source: BMC Surg. 2020 Oct 7;20:229. doi: 10.1186/s12893-020-00889-3 (PMC7542718; doi:10.1186/s12893-020-00889-3)
Supplement: Supplementary file 1 — Additional file 1. [file 12893_2020_889_MOESM1_ESM.pdf]

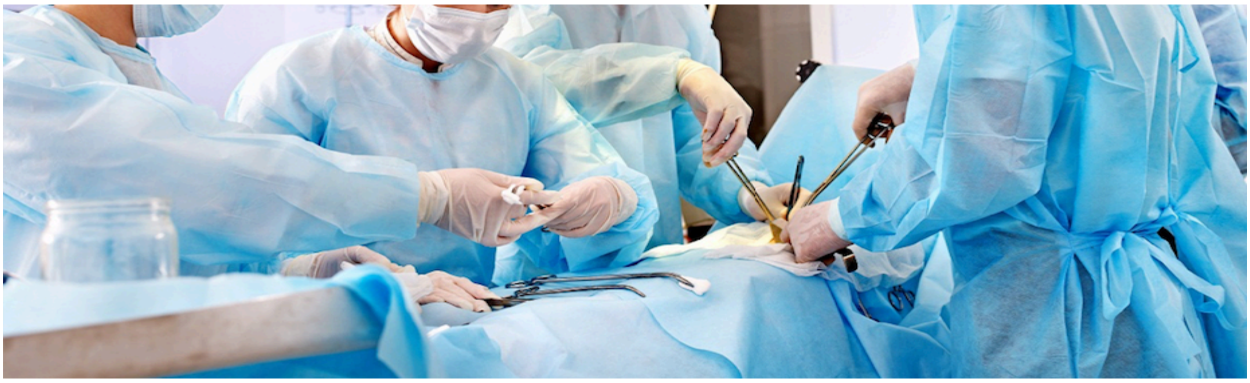

Source: www.google.com

SmartPhone-App for medico-surgical monitoring in post-surgical patients

Thank you for your participation in this study. Participation is voluntary and includes completing an online/paper questionnaire (approx. 5 min). After completion of the data collection, the data are exported to a statistical software and the online survey is deleted. No traceability will be possible from this point onwards. No financial compensation is provided. The data are not passed on to third parties and analyzed anonymously only within the framework of this study. By completing the survey, you give your consent. If you have any questions, do not hesitate to contact me at the following e-mail address:

julian.scherer@uzh.ch

Best regards from Zurich

Cand. med. Julian Scherer

© Julian Scherer

Cand. med. Julian Scherer

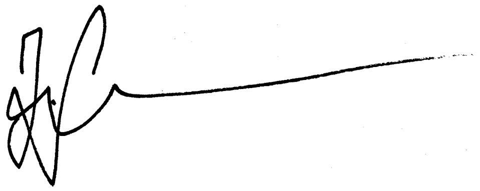

Survey

Scenario:

You are operated by a surgical physician (type / reason of operation is irrelevant).
You are offered to install a SmartPhone app on your mobile phone to monitor the long-term success of the operation and to intervene in the event of any complications, in the sense of early detection, before you can even feel changes.

When answering the questions, please refer to the following app specifications:

1. Data transmission to a server, which is only connected to the respective clinic with the specific medical team, and can only be viewed by them.
2. No evaluation by third parties.
3. NO sensitive data (e.g., geo data via GPS, etc.) are requested.
4. The app analyzes only fixed data (e.g., step number via gyro data acquisition = without tracking, without GPS profile), which can not be corrupted.
5. The app would "report" MAXIMUM (if at all) once a day and you would have to answer one or two questions.
6. The app is free.
7. The app profile is matched with your clinic data.
8. The data would be used anonymously for study purposes.
9. If you have any problems reported by the app, your treating physician would be in touch with you directly.
10. The patient has NO influence on the data recorded by the app (Placebo / Nocebo Exclusion).
11. The patient can ONLY see the absolute data in the app, but no interpretation of this (exclusion anxiety / hypochondria).

Survey

*compulsory reply

Would you install the app? * single answer

- Yes
- No
- Not sure

If no, why not? multiple-choice

- ☐ „Data protection“
- ☐ „Another app that does not help me“
- ☐ „I can tell by myself when something is wrong“
- ☐ „This is too technical for me“
- ☐ Others

If yes, why? multiple-choice

- ☐ „I have direct benefit through the app, in the sense of early detection “
- ☐ „I like to take part in studies to support medical progress“
- ☐ „With the app i feel safe“
- ☐ „I support the technology in medicine“
- ☐ Others

Your gender? *

- female
- male

Your age? *

____

Your profession? *

____

Comments?

____

____

____

____
